# Novel approaches to CMV after HCT: report from the 27th European Congress of Clinical Microbiology and Infectious Diseases, Vienna, Austria, 22–25 April 2017

**DOI:** 10.4155/fsoa-2018-0013

**Published:** 2018-03-14

**Authors:** Rafael F Duarte, Sue Lyon

**Affiliations:** 1 Hospital Universitario, Puerta de Hierro Majadahonda, Madrid, Spain; 2 Freelance Medical Writer & Editor, London, UK

**Keywords:** cytomegalovirus infection, ECCMID, hematopoietic cell transplantation, immunocompromised antiviral treatment prophylaxis

Cytomegalovirus (CMV) infection is a potentially life-threatening complication after hematopoietic cell transplantation (HCT). The potential availability of novel preventive therapies was therefore an important topic during European Congress of Clinical Microbiology and Infectious Diseases (ECCMID) 2017.

A member of the β-herpesvirus family, CMV is a ubiquitous pathogen that is usually acquired in childhood or early adult life through contact with body fluids. After primary infection, the virus becomes latent and rarely causes symptoms in healthy individuals. However, in immunocompromised hosts, CMV infection can lead to invasive disease in almost any tissue, and represents one of the most common and significant complications after allogeneic HCT.

## The burden of CMV after HCT

Although CMV disease-related mortality has declined due to improvements in diagnosis and the use of pre-emptive antiviral treatment, CMV infection has consistently been reported to adversely affect outcomes in allogeneic HCT recipients admitted to the intensive care unit. It is an independent predictor of both in-hospital mortality and of mortality at 12 months (p = 0.005) in HCT patients who survive intensive care unit admission [1,2].

Professor Andrew Ullman (Germany) explained that the direct effects of CMV infection include tissue-invasive, end-organ disease that may affect the upper and/or lower gastrointestinal system (and may be difficult to differentiate from graft-versus-host disease [GvHD]), pneumonia, the central nervous system and the liver and other vital organs [3,4]. The indirect effects of CMV are the consequence of the immunosuppressive and inflammatory effects of the virus on the host’s immune response, and may include acute and extensive chronic GvHD, mortality due to bacterial and fungal infections, transplant-related mortality and lower overall survival [3].

The adverse direct and indirect effects of CMV infection are reflected in healthcare costs, Professor Ullman noted. He described a study in the USA that compared post-transplant treatment costs in 124 HCT recipients, 119 (89%) of whom were at risk of CMV due to donor or recipient seropositivity. CMV reactivation was diagnosed in 90 (75.6%) of these patients at a median of 30 days (range 8–105) post-transplant. Compared with patients who did not require pre-emptive treatment, the treated group incurred additional costs of antiviral medication and longer hospital stays of $58,000–74,000 (€52,000–66,000) per patient within the first 6 months of HCT [5].

## CMV risk factors

According to Professor Jan Styczynski (Poland), the most important risk factor for CMV infection after allogeneic HCT is the serological status of the donor and recipient. Without prophylaxis, about 80% of CMV-seropositive patients develop CMV reactivation, and about 30% of seronegative recipients with a seropositive donor develop primary infection. Other risk factors for CMV infection after allogeneic HCT include treatment with high-dose corticosteroids, T-cell depletion, acute and chronic GvHD and the use of mismatched or unrelated donors [6–8].

In the past, CMV infection was most frequently diagnosed between engraftment and day 100. Following the introduction of pre-emptive antiviral therapy, the prevalence of early CMV disease declined. However, HCT recipients are now increasingly at risk of CMV infection in the later post-transplant period. According to Professor Styczynski, this is a cause for concern, since patients may now no longer be under the care of their specialist center when they develop CMV infection and this may lead to an increased risk of CMV disease later after HCT [7].

The risk of late CMV reactivation is known to be higher in patients with delayed CMV-specific T-cell immune reconstitution following HCT [8]. During ECCMID 2017, Professor Roy Chemaly (USA) presented early results of the REACT study, an ongoing, multicenter, prospective, observational study including adult CMV seropositive allogeneic HCT recipients. T-cell responses are serially monitored pretransplant (screening), and every 2 weeks post-HCT for up to 26 weeks with an ELISPOT assay that uses CMV-specific IE-1 and pp65 antigens [9].

Based on a positive change in spot counts (SPCs) for both IE-1 and pp65 (defined as week 4 SPCs greater than pretransplant SPCs), the negative predictive value for the development of first CMV reactivation was 80.0 and 83.7%, respectively. According to Professor Chemaly, while these results are preliminary, they suggest that assessing CMV-specific cell-mediated immunity at screening and 4 weeks post-HCT may in future help to guide personalized management by determining the degree of protection against CMV reactivation [9].

## Current approaches to CMV management

Isolation of CMV by standard or rapid-culture techniques is now rarely used for monitoring of HCT patients. Guidelines recommend using quantitative nucleic acid testing (most commonly DNAemia), followed by initiation of pre-emptive antiviral therapy at first detection of CMV reactivation or primary infection [4]. Professor Chemaly commented that there is a continuing debate about the threshold for treatment initiation [10], but reminded delegates that after controlling for use of pre-emptive therapy, any level of CMV reactivation increases the risk of overall and early-relapse mortality in the first year after HCT [11].

Intravenous ganciclovir is currently first line for both pre-emptive treatment and prophylaxis of CMV in HCT recipients. Ganciclovir and its oral prodrug valganciclovir are acyclic guanosine analogs, activated via a multistep triphosphorylation process catalyzed by the UL97-encoded viral kinase. Professor Michael McVoy (USA) reported that, when used in prophylaxis, ganciclovir reduces the risk of CMV infection and disease compared with placebo, but does not improve overall survival [6,12].

Foscarnet is a second-line treatment generally reserved for patients unresponsive to ganciclovir because of drug resistance. It is a pyrophosphate analog that inhibits viral DNA polymerase by binding to the pyrophosphate-binding site. Cidofovir is an alternative second-line therapy that is generally used for treatment-resistant CMV strains. It is a competitive inhibitor of viral DNA polymerase, resulting in early chain termination during DNA synthesis [12].

Professor McVoy noted that all antiviral drugs currently used for pre-emptive and prophylactic treatment of CMV target DNA polymerase in order to inhibit DNA synthesis. These drugs are also all associated with significant toxicities, including myelosuppression (ganciclovir) and nephrotoxicity (foscarnet and cidofovir). In his view, such toxicities make these drugs unsuitable for prophylaxis except for high-risk HCT patients, but novel therapies – some with different modes of action – are in development and on current evidence appear to be associated with less toxicity.

## Novel antiviral drugs

Brincidofovir is an oral prodrug of cidofovir. When given twice weekly as CMV prophylaxis in a double-blind dose-escalation study, the most common adverse event associated with brincidofovir was diarrhoea; there were no reports of myelosuppression or nephrotoxicity [13]. In the Phase III SUPPRESS trial, there were fewer clinically significant infections in brincidofovir-treated patients compared with the placebo group at the end of week 14 post-HCT (24 vs 38%; p = 0.002). However, despite this antiviral effect, the study did not meet its primary end point of a reduction in clinically significant CMV infection at week 24 [14].

According to Professor Johan Maertens (Belgium), the failure of SUPPRESS might be due to difficulty in differentiating between diarrhea associated with brincidofovir and the symptoms of GvHD. An intravenous formulation, which has the potential to reduce the likelihood of gastrointestinal adverse effects, is now in early development for CMV prophylaxis and treatment [15].

Maribavir is an oral antiviral drug that prevents exit of new CMV virions from the nucleus by selectively inhibiting UL97-mediated phosphorylation of nuclear Lamin A/C [12]. In a Phase II dose-ranging study, maribavir reduced the incidence of CMV infection in the first 100 days after HCT compared with placebo. The drug was also well tolerated with no evidence of myelosuppression [16]. However, in the placebo-controlled Phase III study, maribavir 100 mg twice daily started after engraftment did not prevent CMV disease in HCT recipients [17].

Professor Maertens considered that the explanation for the negative outcome of the Phase III maribavir study might lie in the use of the rigorous end point of CMV disease in an era of pre-emptive antiviral treatment. There is also the possibility that a higher dose of maribavir might be needed for effective CMV prophylaxis. Maribavir is now being investigated in the pre-emptive treatment of CMV infection in HCT and solid-organ transplant recipients [18].

Letermovir is a terminase inhibitor with a unique mechanism of action. It interacts with the pUL56 viral terminase subunit complex, preventing cleavage of long DNA concatemers and leading to production of noninfectious particles [12]. In the recently reported Phase III trial, letermovir 480 mg once daily (240 mg once daily for patients taking ciclosporin), started by day 28 post-transplant and up to week 14, significantly reduced the risk of clinically significant CMV infection compared with placebo (37.5 vs 60.6%; p < 0.0001) in adult CMV seropositive allogeneic HCT recipients. All-cause mortality at week 24 was 10.2% for letermovir-treated patients and 15.1% in the placebo group [19].

During ECCMID 2017, Professor Chemaly reported in detail on the safety and tolerability of letermovir in the Phase III study. There was no evidence of letermovir-associated myelotoxicity or nephrotoxicity, and there was no difference between letermovir and placebo in the risk of GvHD, diarrhea, nausea, pyrexia, rash and vomiting. Adverse events more commonly seen in letermovir-treated patients compared with placebo were cardiac disorders (12.6 vs 6.3%), ear/labyrinth disorders (4.6 vs 1.0%), hyperkalemia (7.2 vs 2.1%), myalgia (5.1 vs 1.6%) and dyspnea (8.0 vs 3.1%). Most of these adverse events were mild or moderate, were not treatment related as assessed by investigators and did not lead to treatment discontinuation [20].

Letermovir has been granted orphan designation by the European Medicines Agency, the US FDA and the Japanese Ministry of Health, Labor and Welfare for the prevention of CMV infection and disease in at-risk populations. The drug also has been granted Fast Track designation by the FDA.

## Vaccines & antibodies

ASP0113 is a bivalent CMV DNA vaccine consisting of two plasmids, VCL-6368 and VCL-6365. It is currently in clinical trials involving allogeneic HCT recipients, kidney transplant recipients and dialysis patients. Professor Maertens reported that first results are expected in late 2017 of a global Phase III study in CMV-seropositive allogeneic HCT recipients (NCT01877655).

Modulation of the immune system’s ability to manage CMV is an attractive option, and human CMV-specific monoclonal antibodies are now being investigated for their potential in CMV treatment. CSJ148 is a combination of two anti-CMV human monoclonal antibodies (LJP538 and LJP539) that bind to and inhibit the function of viral CMV glycoprotein B and the pentameric complex. When administered to healthy volunteers, CSJ148 was safe and well tolerated with expected pharmacokinetics [21]. A Phase II study has been completed in HCT recipients (NCT02268526).

## Conclusion

Although the risk of CMV disease has fallen following allogeneic HCT, patients remain at risk of the direct and indirect effects of infection with the virus. The toxicity of current antiviral drugs means that CMV prophylaxis is at present limited to high-risk patients, and most HCT recipients receive pre-emptive therapy initiated when CMV infection is detected. The development of less toxic therapies may herald a future change in clinical practice from pre-emptive therapy to prophylaxis of CMV and its complications in HCT recipients and other at-risk groups of patients.
